# The accumulation of particles in ureteric stents is mediated by flow dynamics: Full-scale computational and experimental modeling of the occluded and unoccluded ureter

**DOI:** 10.1063/5.0083260

**Published:** 2022-05-05

**Authors:** Ali Mosayyebi, Aravinthan Vijayakumar, Maryam Mosayebi, Dirk Lange, Bhaskar K. Somani, Costantino Manes, Dario Carugo

**Affiliations:** 1 Department of Mechanical Engineering, Faculty of Engineering and Physical Sciences, University of Southampton, Southampton SO17 1BJ, United Kingdom; 2 Institute for Life Sciences (IfLS), University of Southampton, Southampton SO17 1BJ, United Kingdom; 3 Department of Urologic Sciences, Faculty of Medicine, University of British Columbia, Vancouver V6H 3Y8, Canada; 4 Department of Urology, University Hospital Southampton NHS Trust, Southampton SO16 6YD, United Kingdom; 5 Department of Environment, Land and Infrastructure Engineering, Politecnico di Torino, Turin 10129, Italy; 6 Department of Pharmaceutics, UCL School of Pharmacy, University College London (UCL), London WC1N 1AX, United Kingdom

## Abstract

Ureteric stents are clinically deployed to restore urinary drainage in the presence of ureteric occlusions. They consist of a hollow tube with multiple side-holes that enhance urinary drainage. The stent surface is often subject to encrustation (induced by crystals-forming bacteria such as *Proteus mirabilis*) or particle accumulation, which may compromise stent's drainage performance. Limited research has, however, been conducted to evaluate the relationship between flow dynamics and accumulation of crystals in stents. Here, we employed a full-scale architecture of the urinary system to computationally investigate the flow performance of a ureteric stent and experimentally determine the level of particle accumulation over the stent surface. Particular attention was given to side-holes, as they play a pivotal role in enhancing urinary drainage. Results demonstrated that there exists an inverse correlation between wall shear stress (WSS) and crystal accumulation at side-holes. Specifically, side-holes with greater WSS levels were those characterized by inter-compartmental fluid exchange between the stent and ureter. These “active” side-holes were located either nearby ureteric obstructions or at regions characterized by a physiological constriction of the ureter. Results also revealed that the majority of side-holes (>60%) suffer from low WSS levels and are, thus, prone to crystals accumulation. Moreover, side-holes located toward the proximal region of the ureter presented lower WSS levels compared to more distal ones, thus suffering from greater particle accumulation. Overall, findings corroborate the role of WSS in modulating the localization and extent of particle accumulation in ureteric stents.

## INTRODUCTION

The urinary tract is a critical system of the human body, since one of its primary functions is to regulate the composition of body fluids, which involves filtration and elimination of waste products.^1^ As a result, physical occlusions of this system can have serious consequences on human health. Common causes of obstruction include tumors, kidney stones, and strictures. In these situations, clinicians often deploy a ureteric stent (i.e., a hollow tube with multiple side-holes along its length) to bypass the obstruction and maintain adequate urinary drainage.^2^ Despite being an effective temporary solution against urinary obstructions, stents are often associated with several complications. These include (i) blockage of the stent lumen and/or its side-holes (herein also referred to as ‘‘holes”), which is mainly caused by nucleation, deposition, and accumulation of crystals, and (ii) urinary reflux due to the inability of the ureterovesical junction (UVJ) to fully close in the presence of a stent. Notably, encrusted ureteric stents are often no longer functional as their drainage performance is seriously compromised.^3^ In order to gain a more pervasive understanding of the mechanisms underpinning stent encrustation, theoretical and experimental models have been previously developed to investigate the flow dynamics in the upper urinary tract both in the presence and absence of ureteric stents.^4^ The characteristics of these models are comprehensively described and compared in a recent review article by Zheng and co-authors.^4^

In 1970, Lykoudis and Roos^5^ developed a fluid mechanical model to study the function of the healthy ureter. By imposing physiologically relevant boundary conditions, they demonstrated that the theory of lubrication was able to accurately describe the flow dynamics in the ureter to the extent that the Reynolds number was ∼1. Notably, they designed an approximate equation for urine flow and identified a universal relationship among maximum pressure, urine flow rate, and kinematic behavior of the ureter. It was found that pressure in the ureter during contraction increased by 25 mm Hg compared to its resting state. Reflux or other pathological conditions (i.e., obstructions) were, however, not modeled in their study. Cummings *et al.*^6^ employed theoretical (e.g., mathematical) fluid dynamics to study the effect of a ureteral stent on the flow field within the ureter with a specific focus on reflux. They built an axisymmetric model of the stent to also evaluate the effect of stent side-holes on drainage performance. Due to the loss of peristaltic activity in a stented ureter,^7^ urinary flow was assumed steady and driven solely by the pressure gradient between the upper (kidney pelvis) and lower (bladder) urinary tract. Using this model, it was concluded that reflux due to increased bladder pressure could be reduced by increasing stent porosity (by up to 30%), which could be achieved through the inclusion of side-holes along the length of the stent. Tong *et al.*^8^ carried out a numerical, computational fluid dynamics (CFD) study to investigate the effect on urinary flow of (i) the number of side-holes and their location along the circumference of the stent, (ii) the distance between stent and ureter walls, and (iii) the presence of a ureteric obstruction. The study revealed that, in the absence of ureteric obstructions, side-holes had a very limited effect on the overall urinary flow field. This observation was consistent across models with a differing number, size, and location of ureteric occlusions. The study also demonstrated that fluid exchange between stent and ureter lumens only occurred through side-holes located in proximity to the obstruction (both proximally and distally), which may have implications on particle accumulation and the development of encrustation in these specific regions of the stent. Building on these earlier CFD studies, Kim *et al.* developed a numerical model to investigate urinary flow in the stented ureter, whereby the ureter geometry was varied to encompass tubular, funnel-shaped, and undulated architectures.^9^ The effect of a constriction (or stenosis) of the ureter lumen was also evaluated. It was found that the ureter geometry influenced the distribution and magnitude of urine flow through the stent side-holes. In the tubular ureter, inter-compartmental fluid exchange occurred only through side-holes located in the proximal and distal ureter; conversely, in the funnel-shaped and undulated ureters, flow exchange involved a larger proportion of side-holes. Consistently with Tong *et al.*, in the presence of a ureteric constriction, the role of side-holes was enhanced in close proximity to the constricted region. In two separate CFD studies, they also demonstrated that increasing the number of side-holes throughout the length of the stent resulted in greater urinary drainage (within an undulated ureter model),^10^ while the stent inner diameter inversely correlated with urinary flow rate through the stented ureter.^11^ In a more recent CFD investigation, the same group evaluated the flow performance of stents with differing diameter, using ureter models presenting varying levels of stenosis.^12^ Stents with smaller diameter had superior drainage performance, including within ureter models presenting mild-to-severe stenoses. Further investigations on the function of side-holes and urine flow distribution in the stented ureter have been carried out in a CFD study by Amitay-Rosen *et al.*^13^ Their numerical model replicated either a circumferential occlusion of the ureter or an external pressure applied on one side of the ureter wall. A comprehensive analysis of the urine flow field and pressure levels in the renal compartment was carried out for a range of stent occlusions. Concerning the flow through side-holes, they demonstrated that it is negligible (or very small) in both the unoccluded ureter and in a ureter with 50% encircling obstruction. When a complete obstruction was modeled, the fluid moved into the stent lumen through side-holes upstream of the obstruction and then moved back into the ureter lumen downstream of the obstruction. These findings are consistent with—and further extend—those previously reported by Tong *et al.*^8^ and Kim *et al.*^9^

In these previous models, however, an evaluation of the correlation between stent flow performance and the progression of encrustation or particle accumulation was not carried out. In this regard, a study by Waters *et al.*^14^ employed an axisymmetric theoretical model to investigate urinary flow in a stented ureter and its potential implications on encrustation. As for previous studies, ureteric peristalsis was neglected in their model. Authors concluded that encrustation in ureteric stents does not solely depend on the presence of crystals-forming bacteria (such as *Proteus mirabilis*), but it rather correlates with the physical and chemical characteristics of the local urine environment. It should be noted that numerical CFD models could also be developed to simulate particle dynamics and accumulation in ureteric stents. Upon experimental validation, these models could provide a predictive tool to evaluate particle accumulation in urological devices during pre-clinical development.

In addition to mathematical and computational models, experimental systems have also been developed to investigate the flow field in the stented ureter and its relationship with stent encrustation or particle accumulation. Choong *et al.*^15^ developed a full-scale artificial model of the urinary system that comprised two reservoirs, replicating kidney and bladder compartments, respectively, and a rigid tube replicating the ureter. They utilized this model to assess the rate of encrustation in different commercially available urological stents, consisting of both coated and uncoated materials. Polyurethane was associated with greater encrustation rate compared to silicone, although polyurethane coated with hyaluronic acid overall presented the best anti-encrustation performance. In a more recent study, Clavica *et al.*^16^ developed a polydimethylsiloxane (PDMS)-based model of the obstructed and stented ureter, which was fabricated by replica molding from a 3D-printed master mold. Using this model, they quantified the relationship between renal pressure and urine viscosity, volumetric flow rate, and degree of flow obstruction. The study demonstrated that, at certain clinically relevant boundary conditions and in the presence of a stent, the kidney pressure in the obstructed ureter may increase up to pathological levels. Numerical simulations and experiments were also performed to evaluate the local flow field in close proximity to the obstruction. For the first time, they revealed the presence of laminar vortices within the cavity generated by a ureteric obstruction. These vortices were hypothesized to act as sites where crystals may be trapped and form a nidus for subsequent crystal growth and encrustation; however, the study did not include experimental evidence to validate this hypothesis. In a more recent study, Shilo *et al.* investigated the performance of different stent designs (single lumen, tandem, and metal stents) using an *in vitro* model of the urinary system, comprising a flexible tubing to simulate the ureter and glass vessels to replicate the renal unit and bladder.^17^ A semi-circular compression was applied on the ureter model to replicate an extrinsic ureteral obstruction, and a colloidal solution was employed to mimic the presence of suspended organic and inorganic debris in urine. Results showed that large luminal stents perform better than the other stent designs evaluated in terms of both patency rates and ability to maintain adequate flow of a colloidal suspension. In a follow-up study from the same group,^18^ both computational and experimental modeling were employed to determine the impact of stent diameter and configuration (single vs tandem) on stent failure—which was associated with an increase in renal pressure above a critical value. Notably, in the presence of a complete extrinsic obstruction, the rate of stent failure was independent of either stent diameter or configuration for the same percentage of stent lumen occlusion.

Building upon these earlier investigations, we recently developed a microfluidic-based model of the stented and occluded ureter (referred to as “stent-on-chip”).^19^ Through a combination of experiments and CFD simulations, we revealed that there exists an inverse correlation between the extent of particle accumulation and the magnitude of wall shear stress (WSS) over the stent surface. Moreover, we concluded that the large majority of side-holes in a stent are “inactive” (i.e., they are not subject to inter-compartmental fluid exchange) and are, thus, prone to crystal accumulation, although this was inferred from a “scaled-down” model of the stented ureter. In follow-up studies, we employed this model to also investigate whether specific changes to the design of the stent (including wall thickness and side-hole shape) may promote greater levels of WSS in regions of the stent suffering from greater encrustation rates^20^ and to evaluate the relationship between WSS and bacterial attachment over the stent surface.^21^

Despite previous research has investigated the mechanistic aspects of encrustation development in stents, there appears to be a limited body of work that has attempted to quantitatively correlate urine flow metrics (such as wall shear stress) with the accumulation of encrusting particles within a full-scale model of the stented urinary tract. Therefore, in the present study, we performed a combination of numerical and experimental simulations to assess whether our previous observations in simplified microfluidic-based models are applicable to a more clinically-relevant, full-scale scenario. This would also allow us to assess whether particle accumulation at side-holes is dependent upon their location in the stented ureter, which could not be fully achieved using a microfluidic-based approach. We specifically investigated whether there exists a correlation between particle accumulation and the magnitude of WSS over the stent surface with a focus on the functionality of side-holes. Findings from this study can further our understanding of flow-mediated mechanisms of stent failure, as well as inform the design of stent architectures with increased lifetime.

## RESULTS AND DISCUSSION

### Flow field and particle accumulation in an unobstructed stented ureter model

#### Numerical characterization of the flow field in the unobstructed stented ureter model

**FIG. 1.**
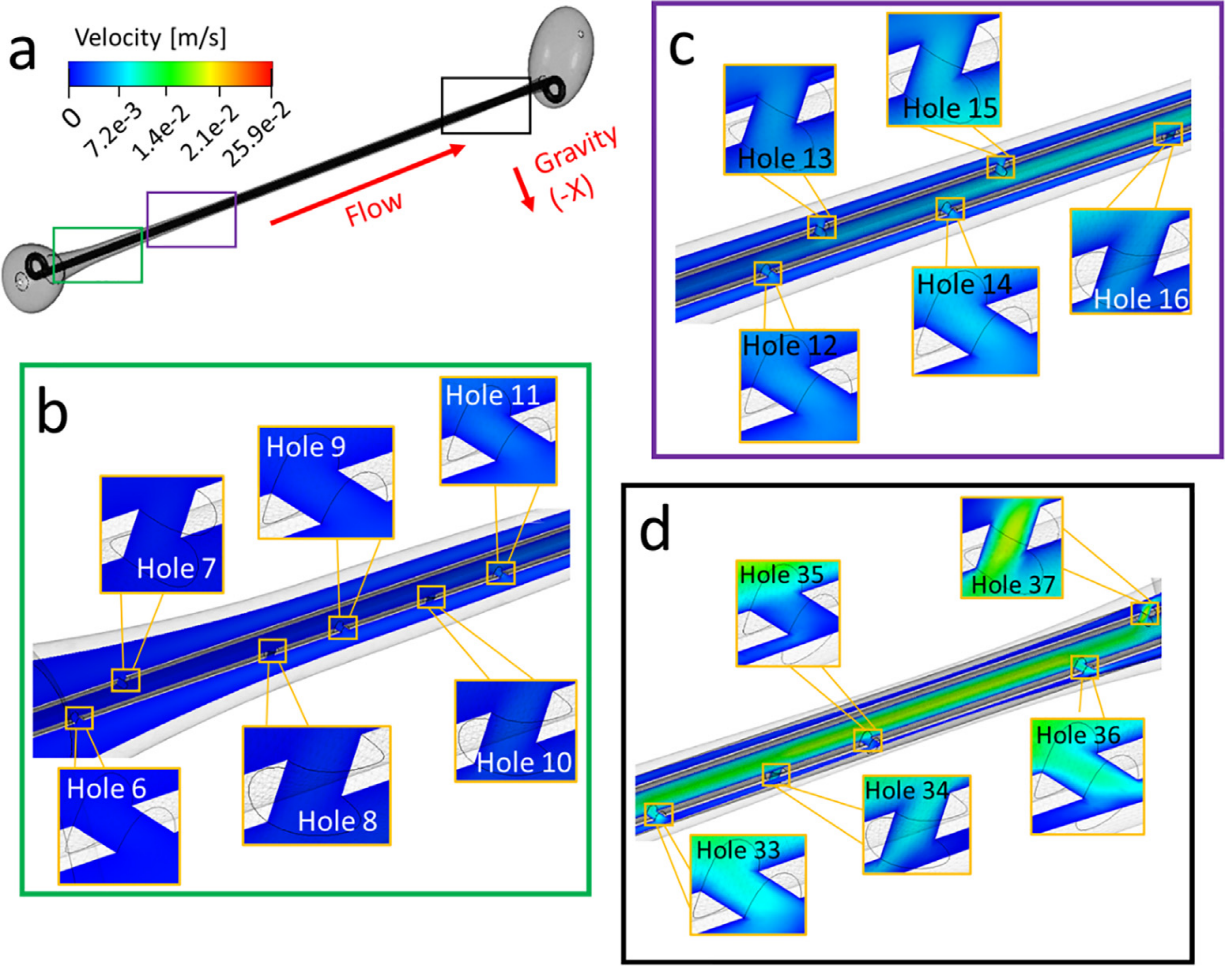
(a) Contours of the velocity magnitude (in m/s) taken at different positions along the model and computed numerically over the model mid-plane. Insets show zoomed-in views at different regions along the ureter (from proximal to distal). These include (b) holes 6–11, (c) holes 12–16, and (d) holes 33–37. Both intra- and extra-luminal compartments nearby a side-hole are shown. Results refer to the unobstructed model.

Figures 1(a)–1(d) show contours of the velocity magnitude determined from CFD simulations of the unobstructed stented ureter model, taken over the model mid-plane. Results show that the maximum velocity magnitude is greater within side-holes located in the distal region of the model [Fig. 1(d)] compared to those located closer to the proximal end of the model [Figs. 1(b) and 1(c)]. In particular, values ranged between 2.6–19.1 and 0.4–5.8 mm/s at side-holes located in the vicinity of the bladder (holes 33–37) and kidney pelvis (holes 6–15), respectively (see supplementary material Table S1 for a list of maximum velocity values at these side-holes). These results, thus, likely indicate that, in the case of an unobstructed and stented ureter, distal side-holes of the stent experience a greater degree of inter-compartmental flow exchange compared to the more proximal side-holes. This may be due to a reduction in the diameter of the ureteral lumen in the region closer to the UVJ (and a corresponding increase in the associated hydraulic resistance), which diverts part of the flow from the ureteral lumen into the stent. These findings are also consistent with earlier numerical investigations showing that, in the absence of occlusions of the ureter or stent, the total flow rate partitions between the stent and ureter lumens on the basis of their relative cross-sectional areas.^13^

**FIG. 2.**
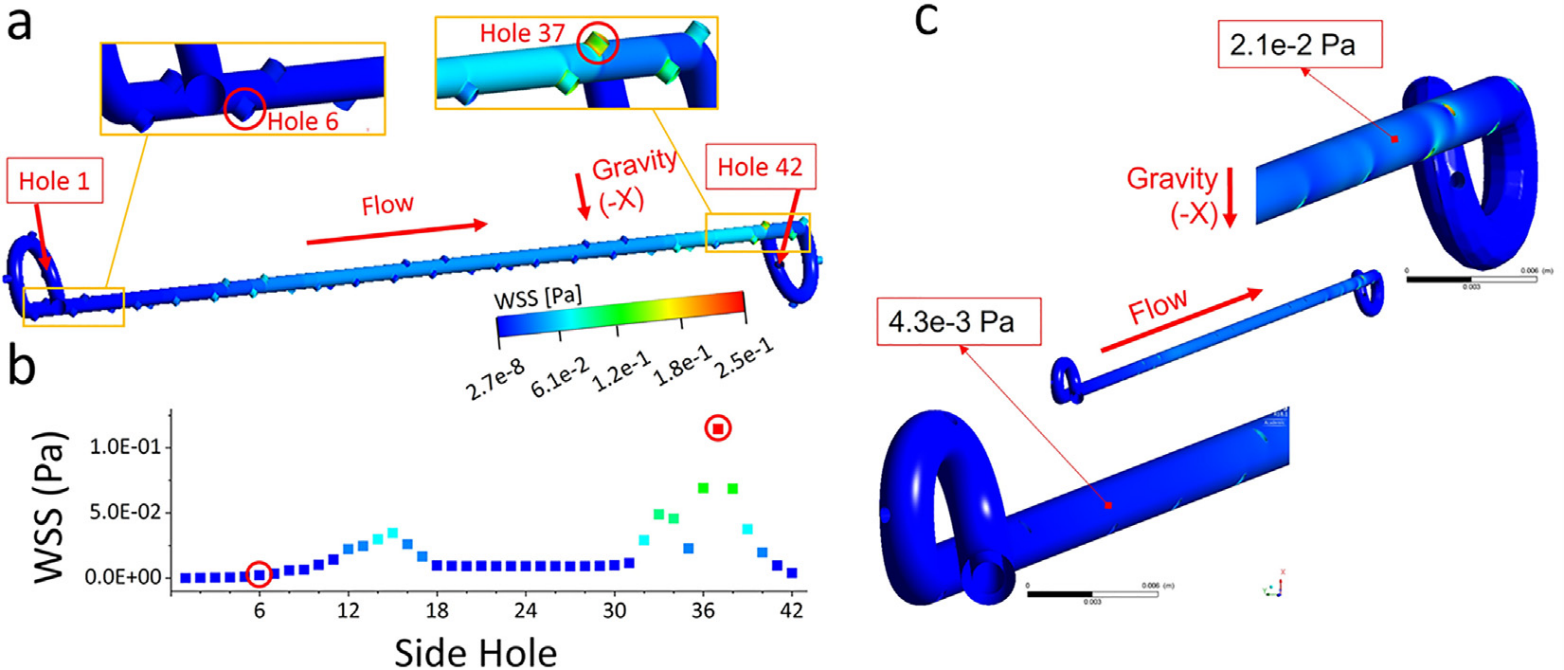
(a) Contours of the WSS magnitude (in Pa) over the stent internal wall and the lateral walls of side-holes, as computed numerically. Zoomed-in views of the WSS contours at both the proximal (holes 1–8) and distal (holes 23–42) regions of the unobstructed and stented model are also shown. (b) Mean values of the WSS magnitude (in Pa) acting over the wall of each individual side-hole from hole 1 to hole 42. Red circles are used to indicate corresponding holes between figures (a) and (b). (c) Contours of the WSS magnitude (in Pa) over the stent external wall, as well as zoomed-in views of the WSS contours at both the proximal and distal regions of the unobstructed model.

Figure 2(a) shows the spatial contours of the WSS magnitude in the unobstructed and stented ureter model, which are determined from numerical simulations and plotted over the stent internal wall and the lateral walls of side-holes. Figure 2(b) instead shows values of mean WSS for each of the stent side-holes (see supplementary material Table S2 for a list of numerical WSS values at these side-holes). Results show that the lowest WSS magnitude corresponds to side-holes located in the renal pelvis, ranging between 3.5 × 10^−5^ and 5.0 × 10^−4^ Pa (holes 1–4). This appears to support earlier qualitative clinical observations, as well as quantitative analyses on stents retrieved from patients, showing enhanced encrustation over the stent coils.^35–37^ The WSS over both the internal wall of the stent and side-holes slightly increased from hole 6 to hole 12, although it was lower than the critical value of 8 × 10^−2^ Pa that, in our previous study, was shown to correlate with increased particle accumulation rates.^19^ It then reduced to about 9.0 × 10^−3^ Pa until hole 31 and increased only in the more distal region, ranging between 2.3 × 10^−2^ and 6.9 × 10^−2^ Pa (at holes 33–36). The distal coil of the stent also suffered from low WSS levels, as for the proximal coil, which supports observations of enhanced encrustation at both coils of the stent.^37^ The box plot in supplementary material Fig. S1 shows that the maximum, minimum, and mean WSS magnitude over the internal wall of the stent were 1.4 × 10^−2^, 2.0 × 10^−5^, and 2.0 × 10^−3^ Pa, respectively. Taken together, the spatial distribution of the WSS and velocity magnitude confirms that side-holes characterized by inter-compartmental flow exchange (herein referred to as active side-holes) experience greater levels of WSS, coherently with the findings from our previous study using a microfluidic-based model.^19^ Moreover, the magnitude of WSS at side-holes in this full-scale study is within the range of values determined using our previously reported stent-on-a-chip model,^19^ which further corroborates the validity of a microfluidic-based approach. Overall, flow exchange through side-holes predominately occurred in regions of the model that were characterized by a reduction of the ureter lumen (mainly in proximity to the UVJ). Flow at these side-holes was mainly directed into the stent, resulting in a corresponding increase in the WSS acting over the inner surface of the stent. Notably, the majority of side-holes experienced WSS levels that are anticipated to favor significant accumulation of crystals and may, therefore, suffer from partial or complete occlusion.^19^ Previous computational studies have also consistently shown that a significant proportion of side-holes is not involved in flow exchange between the stent and ureter;^12,18,38^ however, the correlation between WSS levels and particle accumulation at side-holes within a full-scale model of the stented ureter has not been previously investigated.

Concerning the WSS acting over the stent external wall [Fig. 2(c) and supplementary material Fig. S2], the maximum, minimum, and mean values were equal to 1.1 × 10^−1^, 1.2 × 10^−6^, and 1.3 × 10^−2^ Pa, respectively. Importantly, values were lower than those observed over the internal wall of the stent, at corresponding locations, suggesting that the external surface of the stent may be more susceptible to particle accumulation. This could be due to changes in the diameter of the ureter lumen along its length (for a constant stent diameter), leading to fluid exchange through side-holes being mainly directed into the stent lumen (as shown in Fig. 1). These findings appear to support an earlier *in vivo* study by Brewer *et al.* on a stented porcine model,^39^ which found greater levels of intra-luminal flow when compared to the extra-luminal ones.

#### Particle accumulation at side-holes inversely correlates with wall shear stress

**FIG. 3.**
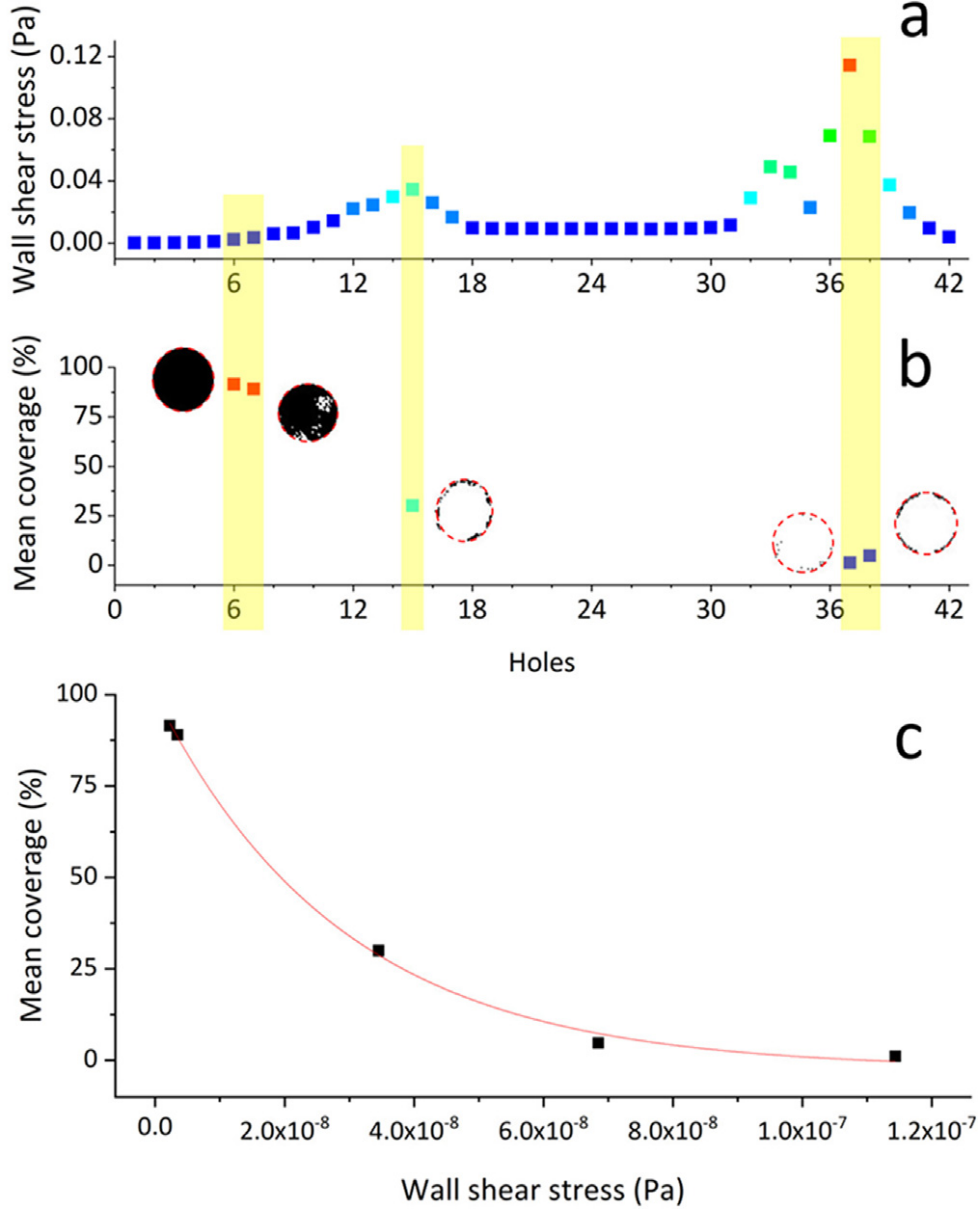
(a) Mean values of the WSS magnitude (in Pa), calculated numerically for each individual side-hole (from hole 1 to hole 42). (b) % coverage area occupied by crystals at side-holes located in specific regions of interest within the model (i.e., hole 6, hole 7, hole 15, hole 37, and hole 38). The end point binarized images of particles accumulated at side-holes are also reported next to the corresponding coverage area data points. Black pixels in the images correspond to the presence of particles. (c) Plot of the mean coverage area (in %, measured experimentally) as a function of the mean wall shear stress (in Pa, determined numerically) taken at selected side-holes of the stent (corresponding to holes 6, 7, 15, 37, and 38). The red line corresponds to a non-linear (exponential) fit of the data points.

In order to evaluate whether there exists a correlation between WSS and particle accumulation in a full-scale model of the stented ureter, an experiment was conducted employing the ureter model shown in Fig. 8. Figure 3(b) shows the mean % area covered by accumulated crystals, taken at selected side-holes of the stent. Measurements were performed at different regions along the model, corresponding to different levels of WSS in the simulations [shown in Fig. 3(a)]. Numerical values of mean WSS and % coverage area are also reported in Table S3. Notably, a non-linear inverse correlation was determined between WSS at side-holes and % coverage area, as shown in Fig. 3(c). This is consistent with the findings reported in our previous study using a simplified microfluidic-based model.^19^ Notably, when WSS increased from 2.2 × 10^−3^ (hole 6) to 1.1 × 10^−1^ Pa (hole 37), the corresponding mean coverage area significantly reduced from 91.5% to 1.1%. Results also indicate that the large majority (>60%) of side-holes likely suffer from significant accumulation of crystals with only a limited number of active side-holes remaining unobstructed.

**FIG. 4.**
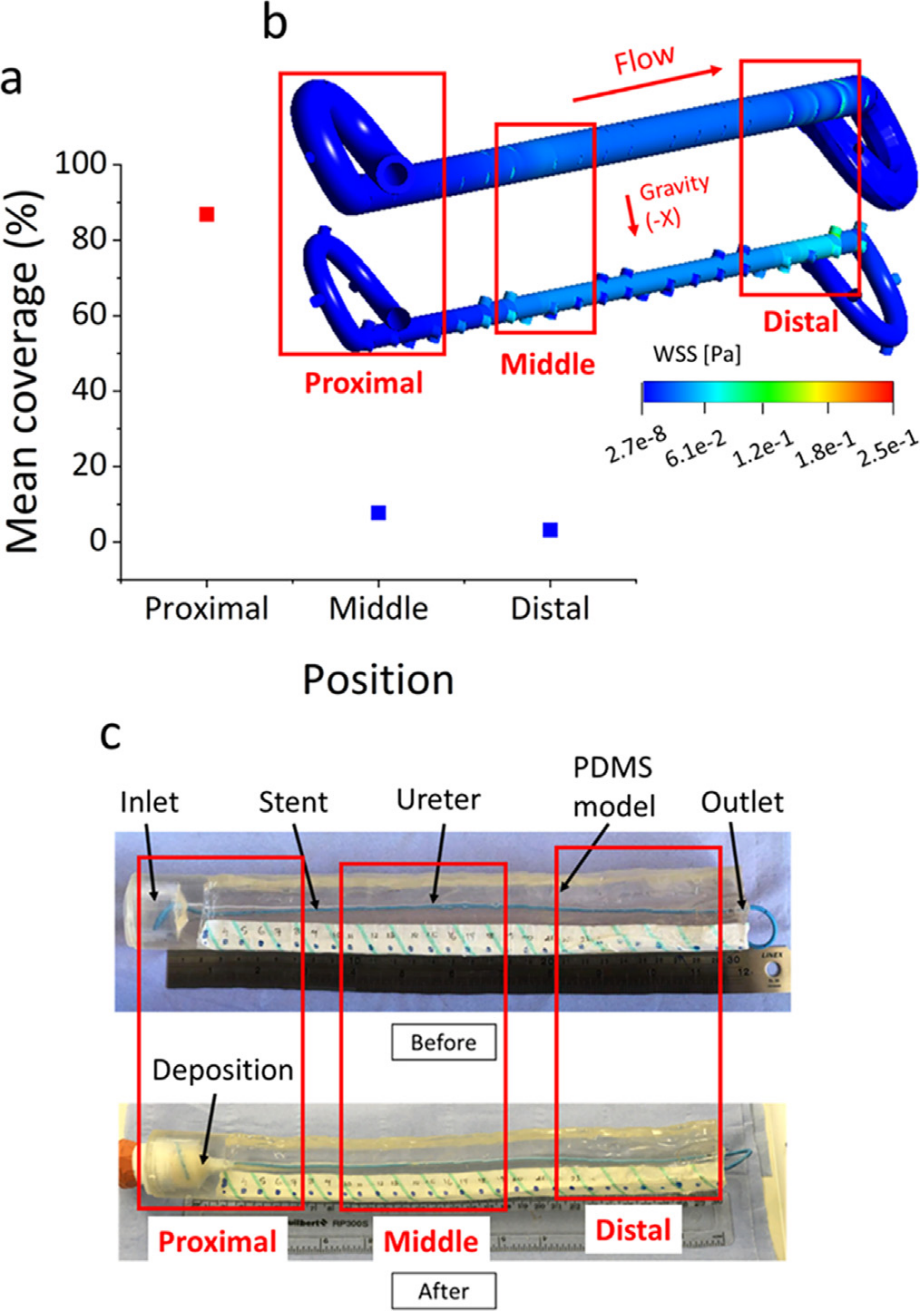
(a) The mean % coverage area occupied by accumulated particles, quantified as an average of values taken at side-holes located in specific regions (or domains) of interest within the model (i.e., defined as proximal, middle, or distal). (b) Contours of the WSS magnitude over the outer (top) and inner (bottom) surface of the stent. Red boxes indicate the corresponding regions of interest over which the mean % coverage area was quantified. (c) The top view of the model before and after particle-accumulation experiments with indicated the regions over which the mean % coverage area was quantified.

Figure 4 shows the mean % coverage area at side-holes, reported as an average over a specific spatial domain of the model; i.e., proximal, middle, or distal. The relative location and length of these domains are illustrated in Fig. 4(c). Consistently with the results shown in Fig. 3, side-holes of the stent characterized by low WSS levels presented a greater amount of accumulated particles [see Fig. 4(b)]. Proximal, middle, and distal domains had 87.0%, 7.7%, and 3.2% coverage area inside side-holes, respectively, and a mean WSS of 3.4 × 10^−5^, 3.5 × 10^−2^, and 1.1 × 10^−1^ Pa. These findings, thus, suggest that the proximal segment of a ureteric stent may be more susceptible to failure due to the accumulation of crystals.

**FIG. 5.**
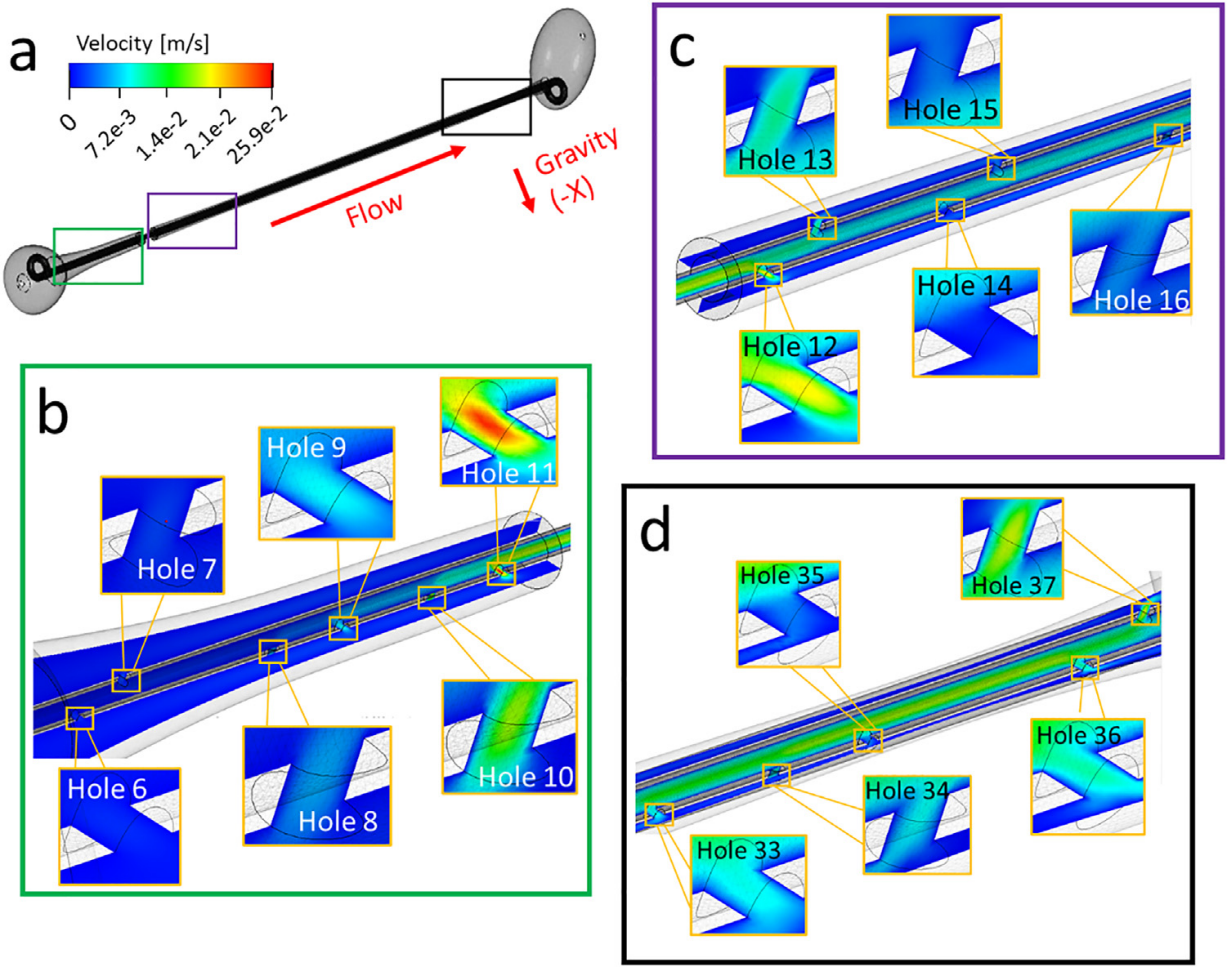
(a) Contours of the velocity magnitude (in m/s) computed numerically over the model mid-plane. Insets show zoomed-in views of the contours at different regions along the ureter (from proximal to distal). These include (b) holes 6–11, (c) holes 12–16, and (d) holes 33–37. Both intra- and extra-luminal compartments nearby a side-hole are shown. Results refer to the obstructed model.

In summary, our findings using a full-scale unobstructed and stented ureter model demonstrate an inverse correlation between the WSS magnitude (determined from CFD simulations) and the extent of particle accumulation. It was also shown that the large majority of side-holes in a stent are inactive and are, thus, prone to particle accumulation, and this is more enhanced for side-holes located toward the proximal region of the stented ureter. These findings concur with recent observations by Zheng and co-authors on stents retrieved from stone patients, which were analyzed using micro-computed tomography (*μ*CT) and image segmentation to quantify the volume of stent encrustation.^37^ Using this method, they revealed that side-holes often act as anchoring sites for encrustation (even for stents with short indwelling times), and that the proximal stent suffers from greater levels of encrustation compared to the distal stent. When combined with findings from the present study, these observations may, therefore, suggest a role for WSS in modulating the localization and amount of mineral crystals in stents.

In a subsequent phase of the study, the flow field in an obstructed model of the stented urinary system was investigated.

### Flow field in an obstructed stented ureter model

The CFD model was employed to investigate the effect of a ureteric obstruction on the WSS field acting over the stent surface. Figures 5(a)–5(c) show the contours of the velocity magnitude plotted over the model mid-plane, while supplementary material Table S4 reports a list of maximum velocity values at selected side-holes. Similar to the unobstructed model, results show greater velocity magnitude at side-holes located at the distal region of the model (ranging from 2.6 to 18.9 mm/s, at holes 33–37) compared to those located at the proximal region. Greater velocity magnitude at side-holes located just before and after the obstruction was also observed and was equal to 27.5 mm/s at hole 11 (before the obstruction) and 22.2 mm/s at hole 22 (after the obstruction). Results, thus, show that there is a greater degree of flow exchange through side-holes located in the vicinity of a ureteric obstruction, allowing the urine to by-pass the source of obstruction, and also in correspondence to the physiological narrowing of the ureter lumen located nearby the UVJ (as for the unobstructed model). These observations are consistent with those reported in earlier modeling studies.^12,13,38^

**FIG. 6.**
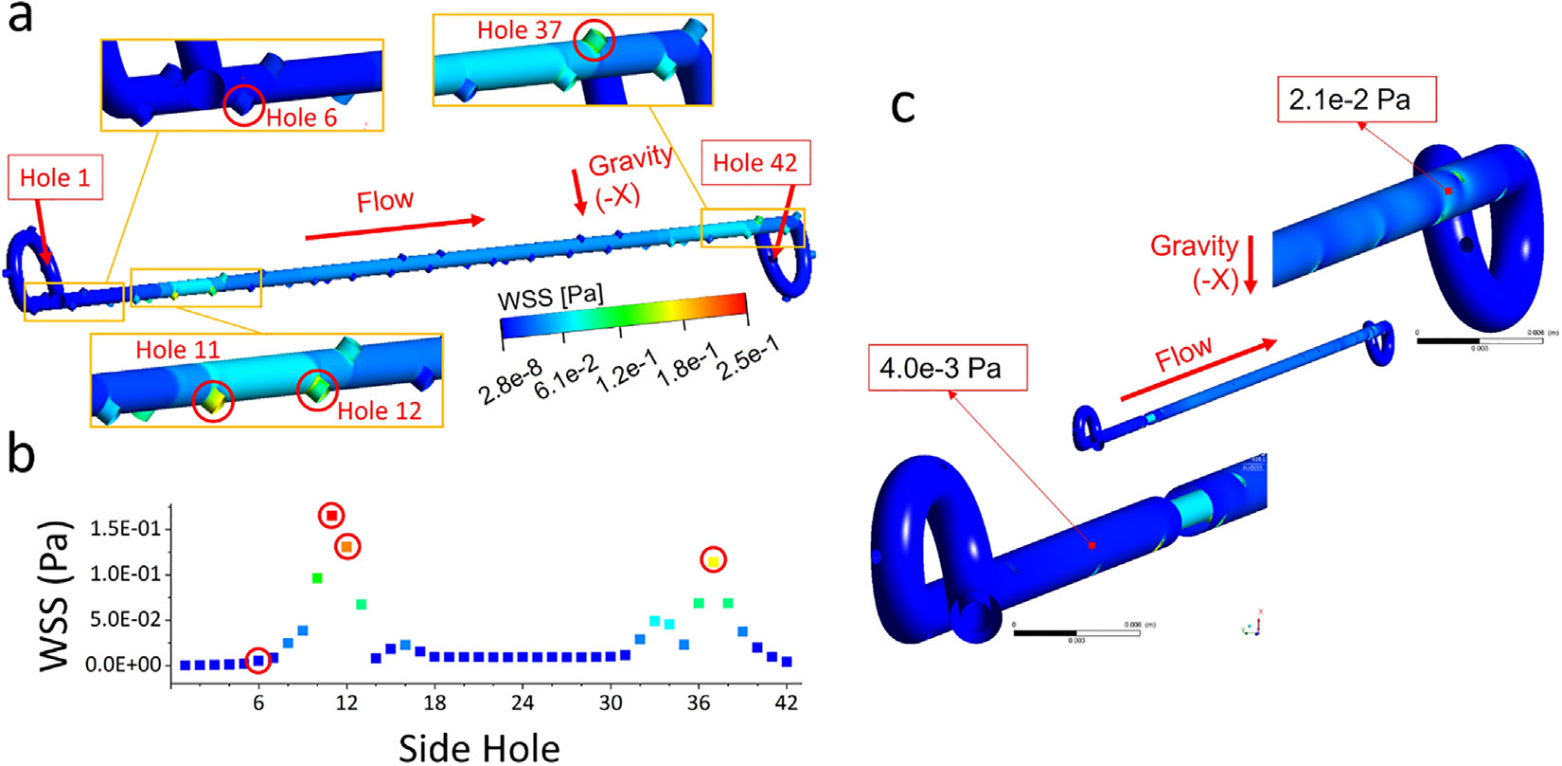
(a) Contours of the WSS magnitude (in Pa) over the stent internal wall, as well as WSS contours for both the proximal (holes 1–8) and distal (holes 23–42) regions (obstructed model). (b) Mean values of the WSS magnitude (in Pa) for each individual side-hole, from hole 1 to hole 42. Red circles are used to indicate correspondence of side-holes between (a) and (b). (c) Contours of the WSS magnitude (in Pa) over the stent external wall and zoomed-in views at both the proximal and distal regions (obstructed model).

Figure 6(a) shows the spatial distribution of the WSS magnitude in the obstructed and stented ureter model, plotted over the stent internal wall. Figure 6(b) instead shows the values of the mean WSS for each of the stent side-holes. (Numerical values are also summarized in supplementary material Table S5.) As for the unobstructed model, the lowest WSS magnitude was at the lateral walls of side-holes located in the kidney pelvis (ranging between 7.1 × 10^−5^ and 1.0 × 10^−3^ Pa, at holes 1–4). The WSS over both the internal wall of the stent and side-holes significantly increased from holes 6 to 18 (located before and after the obstruction) within the range between 5.2 × 10^−3^ and 1.7 × 10^−1^ Pa. It then reduced to about 9.5 × 10^−3^–9.9 × 10^−3^ Pa until hole 31 and increased only in the more distal region (ranging from 2.3 × 10^−2^ to 1.1 × 10^−1^ Pa, at holes 33–37). As for the unobstructed model, the distal coil of the stent suffered from low WSS levels. The box plot in supplementary material Fig. S3 shows that the maximum, minimum, and mean WSS magnitude over the stent internal wall were 1.9 × 10^−1^, 3.0 × 10^−5^, and 2.3 × 10^−2^ Pa, respectively. The WSS and velocity distributions, thus, show that side-holes characterized by inter-compartmental flow exchange (referred to as active side-holes) experience greater levels of WSS, which is coherent with our earlier microfluidic-based findings^19,20^ and with full-scale simulations of the unobstructed model reported above (see Fig. 2). Flow exchange occurred in regions of the model that were characterized by a reduction of the ureter lumen and particularly in proximity to the obstruction and the UVJ.

**FIG. 7.**
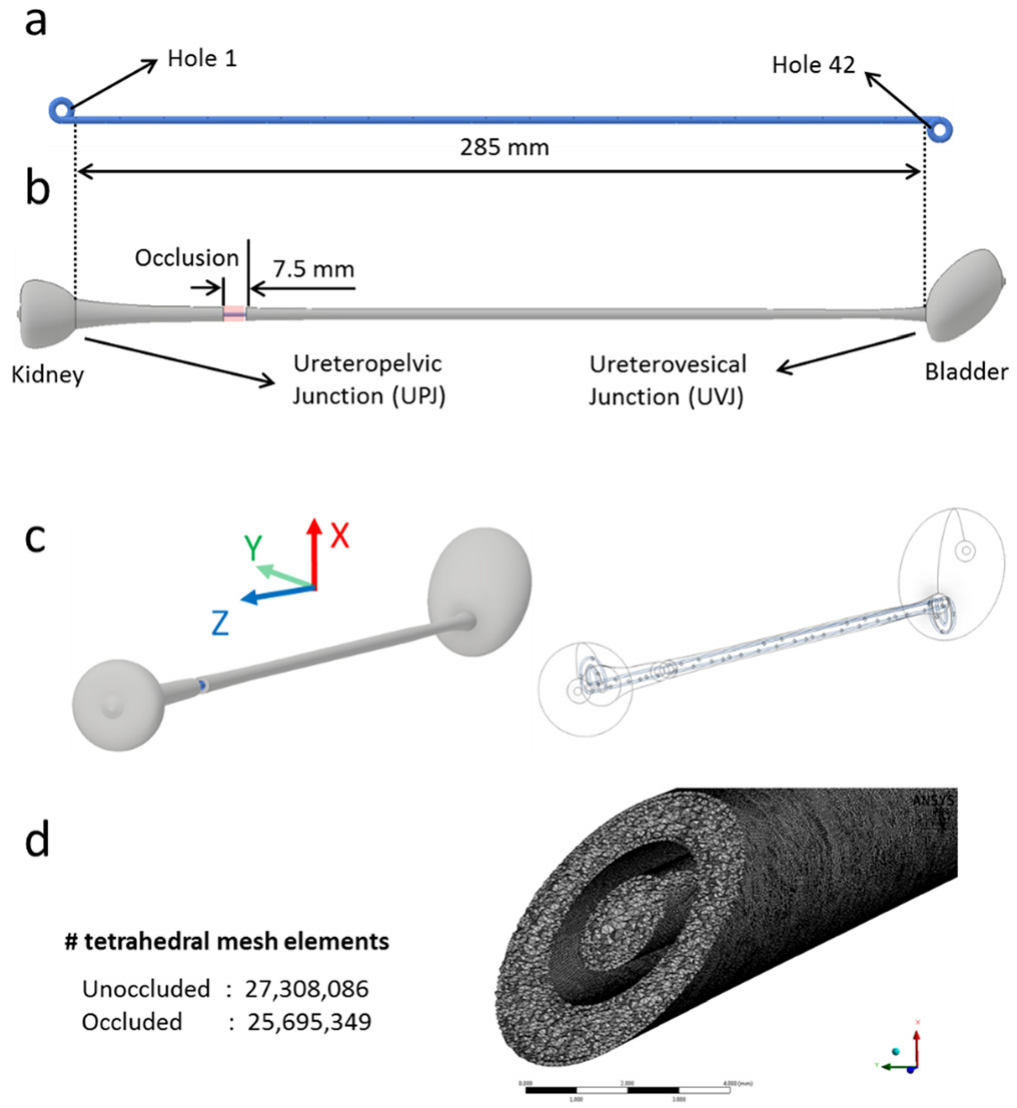
(a) CAD drawing of the stent, where the geometrical properties are taken from the commercial double-J stent UniversaVR (CookVR Medical, USA). The stent has a wall thickness of 0.5 mm, a length of 28.5mm (excluding coils), an internal diameter of 1.5 mm, and a total number of 42 side-holes that are placed at intervals of three to four with an average spacing between side-holes of about 7.5 mm. (b) Model of the urinary system, including kidney, ureter, and bladder compartments. The geometrical properties required to construct this model were taken from Ref. 16. The image also shows the modeled ureteric obstruction (highlighted in red). It was positioned at a distance of approximately 50mm from the UVJ between hole 11 and hole 12. The unobstructed model has the same geometrical characteristics, except for the obstruction. (c) Symmetric view of the stented urinary system (obstructed), including its wireframe view and the corresponding reference coordinate system. (d) Zoomed-in view of the tetrahedral meshing performed on the obstructed model. The number of mesh elements for both unobstructed and obstructed models is also reported.

Figure 6(c) shows the spatial distribution of the WSS magnitude over the stent external wall of the obstructed model, while supplementary material Fig. S4 shows a box plot to illustrate the distribution of WSS values. The maximum, minimum, and mean WSS were 1.8 × 10^−2^, 1.9 × 10^−6^, and 1.3 × 10^−1^ Pa, respectively. As observed in the unobstructed model, values were lower than those acting over the internal wall of the stent at corresponding locations [see Fig. 6(a)], suggesting that the external stent surface may be more susceptible to particle accumulation. As discussed previously, this could be attributed to the effect of variations in the ureteric inner diameter on the urinary flow distribution, overall resulting in greater volumes of urine being diverted into the stent lumen as opposed to the extra-luminal compartment.

In summary, findings obtained using the obstructed model align with those discussed above for the unobstructed model. The primary difference between the two models is in the increased inter-compartmental flow exchange (and corresponding WSS levels) at side-holes located in the close vicinity of the ureteric obstruction. From these findings, it can, therefore, be inferred that—even in the occluded ureter—the majority of side-holes experience low WSS levels and are, thus, likely to suffer from particle accumulation.

### Limitations of the study

The study described in this paper presents several limitations relating to the model and methods employed, which are discussed below. Future work should, therefore, assess whether findings are applicable to conditions that are more physiologically and clinically relevant. (a) The ureter model in this study had a straight centerline, and both stent and ureter were axisymmetric. The physiological ureter instead shows some characteristic curvatures with the stent touching the inner ureter wall in multiple regions. These factors may likely influence flow exchange at side-holes. Based on previous modeling studies,^9,12,13^ it could be anticipated that the number of active side-holes would increase as a result of these physiological curvatures and asymmetries, due to local fluid pressure variations between the stent and ureter lumens driving the fluid into/out of the stent.^13^ Similar considerations apply to the stent geometry, which was modeled as straight in the present study but would also present curvatures once deployed in a patient. (b) The study was conducted with the model oriented horizontally. In a real scenario, however, different orientations would subsist that would merit further investigation. Although it is not anticipated that values of WSS would undergo significant changes with varying the model orientation, the latter could impact on the spatial localization of particles due to the effect of gravitational forces on particle's trajectory. For instance, in the case of a vertical orientation, increased particle accumulation in the more distal side-holes may potentially occur. These effects would, however, be dependent on particle's size and density, among other factors. Future work could, thus, focus on a more in-depth characterization of the physical and dimensional properties of the formed crystals in order to gain an even more pervasive understanding of the factors governing particle accumulation. (c) The flow field was assumed to be stationary, and the ureter/stent walls were assumed rigid, as both ureteric peristalsis and bladder micturition were not modeled in this study. This also implied neglecting the occurrence of urinary reflux, which previous studies have indicated as a potential contributing factor to the observed greater encrustation levels in the proximal stent.^37^ A time-dependent computational model to account for these effects could be developed in the future work, while a pressure-controlled experimental model could enable replication of reflux. Concerning ureter peristalsis, it should be noted that placement of a double-J stent has been associated with inhibition of peristaltic activity.^40^ (e) The image acquisition method could be improved to enable simultaneous quantification of particle accumulation at each individual side-hole of the stent. Notably, a recent study has demonstrated the potential of *μ*CT as a means to quantify encrustation in stents.^37^ A similar approach could be adopted in the future, which would also address the two-dimensional (2D) nature of the method used herein. Finally, (f) encrustation is a complex multi-step process that depends on several physical, chemical, and micro-biological factors. The present study focused on the accumulation of crystals from a supersaturated urine surrogate. While particle accumulation may exacerbate or enhance encrustation, it is only one of the processes potentially responsible for stent failure. In order to assess whether WSS can modulate the formation and growth of encrustation in stents, future studies could employ a surrogate fluid that more closely replicates the properties of urine (including the presence of urease-producing bacteria). This may also contribute toward isolating the contribution of different processes—i.e., encrustation, biofilm formation, particle accumulation—on stent failure.

## CONCLUSIONS

Ureteric stents are endourological devices consisting of a hollow tube with multiple side-holes along its length and two J-shaped coils at both ends. The demonstrated function of side-holes is to promote urinary drainage in the case of a ureteric obstruction or other pathological conditions. Despite their proven clinical efficacy, a large proportion of stents suffer from encrustation (often occurring at stent side-holes and coils),^37,41^ potentially leading to premature stent failure. Several computational and experimental models have been developed to characterize the flow dynamics in the stented ureter and gain a more comprehensive understanding of the mechanisms of stent failure.^4^ Only few notable studies, however, have focused on correlating the urine flow characteristics with factors that are responsible for stent failure such as encrustation or particle accumulation.^14,17,18^ In a previous investigation, we demonstrated an inverse correlation between wall shear stress (WSS) and particle accumulation in stents using a 2D microfluidic-based model that replicated a short segment of the stented ureter.^19,20^ Given that WSS has the potential to modulate the localization and extent of crystal accumulation, the aim of this study was to evaluate whether our earlier findings could be extended to a more clinically relevant, full-scale model of the stented ureter. This would also allow us to assess whether particle accumulation at side-holes is dependent upon their location on the stent, which could not be achieved using a scaled-down microfluidic model. In order to fulfill these objectives, we developed a 3D full-scale architecture of the urinary system to computationally investigate the flow dynamic performance of a ureteric stent, as well as experimentally evaluate particle accumulation over the stent surface.

The combination of *in vitro* experiments and CFD simulations conducted in this study revealed an inverse correlation between wall shear stress and accumulation of crystals at side-holes of a full-scale stent model. Results also demonstrated that the majority of side-holes (>60%) in a stent suffer from low WSS levels and are, thus, prone to the accumulation of crystals. Moreover, side-holes located toward the proximal region of the ureter present lower WSS levels compared to the more distal ones. Side-holes with greater WSS levels are those characterized by inter-compartmental fluid exchange between the stent and ureter lumens (referred to as active side-holes). These side-holes are located either nearby pathological ureteric obstructions or at regions characterized by a physiological reduction of the ureteral lumen (i.e., close to the UVJ). Previous studies have shown that occlusion of side-holes has only limited impact on renal pressure but also concurred that maintaining side-hole patency is important to facilitate fluid transfer between the stent and ureter lumens in the presence of pathological or physiological constrictions.^18^

Findings from this study, thus, corroborate our earlier microfluidic-based observations^19^ and suggest that WSS could be employed as one of the predictors for particle accumulation in stents and, thus, guide the design of stent architectures with potential for increased lifetime. For example, the shape and diameter of side-holes could be varied in an attempt to favor activation and/or increased WSS levels at side-holes. We have previously reported on a streamlined side-hole architecture that achieves greater levels of WSS compared to a conventional shape.^20^ Further efforts could also be devoted to increase WSS levels locally (i.e., in the proximal ureter and stent coils), for example, through local changes in the number of side-holes or stent diameter in those regions. The developed computational model could be employed to screen the performance of different commercial stent designs against particle accumulation. The model could also be modified to enable investigation of the flow performance of other endourological devices, such as sphincter stents or urethral catheters, which are also known to suffer from encrustation, particularly upon long-term deployment.^42,43^ Previous studies have demonstrated a qualitative dependence of encrustation on the flow field within a urethral catheter;^44^ a more quantitative investigation could, thus, be performed in future research. Numerical models could also be further developed to include simulation of particle dynamics in urological devices. Upon experimental validation, these models could, thus, provide a predictive tool to evaluate particle accumulation in urological devices during pre-clinical development. Finally, renal pressure was not measured in this study and could be quantified in future experiments to evaluate whether it correlates with the deposition of crystals. This follows from previous studies that have investigated this process using colloidal suspensions as a urine surrogate.^29^

## METHODS

### Rationale and design of the study

Encrustation of ureteric stents is a complex and multi-step process. Upon deployment, a stent is rapidly coated with a conditioning layer that comprises different organic molecules (including glycoproteins).^22^ The stent surface may then be colonized by urease-producing bacteria (primarily *Proteus mirabilis*), which results in elevated urine pH levels and subsequent formation of crystalline deposits over the stent surface.^23^ Encrustation could, however, also occur spontaneously (i.e., in the absence of urease-producing bacteria) due to elevated levels of minerals in urine, also resulting in crystal deposition over the stent.^24^ Crystals (and other particles) that are suspended in urine can also accumulate on the stent—a process that is dependent upon the flow dynamic conditions.^18^ This may also further exacerbate the build-up of crystalline biofilms or encrustation,^23^ potentially increasing the rate of stent failure. The focus of the present study is specifically on particle accumulation.

The ureter model used herein—in both numerical simulations and experiments—was based on the one developed by Clavica *et al.*,^16^ which was reconstructed from *ex vivo* pig ureter samples. The ureter diameter in the model decreased from approximately 6.0 to 3.0 mm in the proximal segment (in the vicinity of the ureteropelvic junction, UPJ) and remained almost constant and equal to approximately 2.5 and 2.4 mm in the middle and distal segments, respectively. It then slightly increased to 2.9 mm at the UVJ. It should be noted that previous studies have indicated that there are no significant age and gender differences in ureteric diameter.^25^ Studies have also shown that the anatomy and physiology of the porcine ureter are broadly comparable to the ones of the human ureter,^26^ including its tissue structure^27^ and urodynamic parameters.^28^ For these reasons, the porcine urinary system is often employed in pre-clinical research on ureteric stents.^29^

In the occluded model, a 7.5 mm long complete occlusion of the ureteric lumen was designed in the proximal region of the ureter, consistently with previous *in vitro* studies using artificial ureter models.^16^ Moreover, previous clinical studies have shown that a significant proportion (∼37%) of unilateral ureteral stones lodge within the upper ureter.^30^ The modeled occlusion was positioned at a distance of approximately 50 mm from the UVJ between hole 11 and hole 12. The kidney pelvis and bladder compartments were modeled as two enclosed reservoirs, proximal and distal to the ureter model, respectively [see Figs. 7(a)–7(c)].

**FIG. 8.**
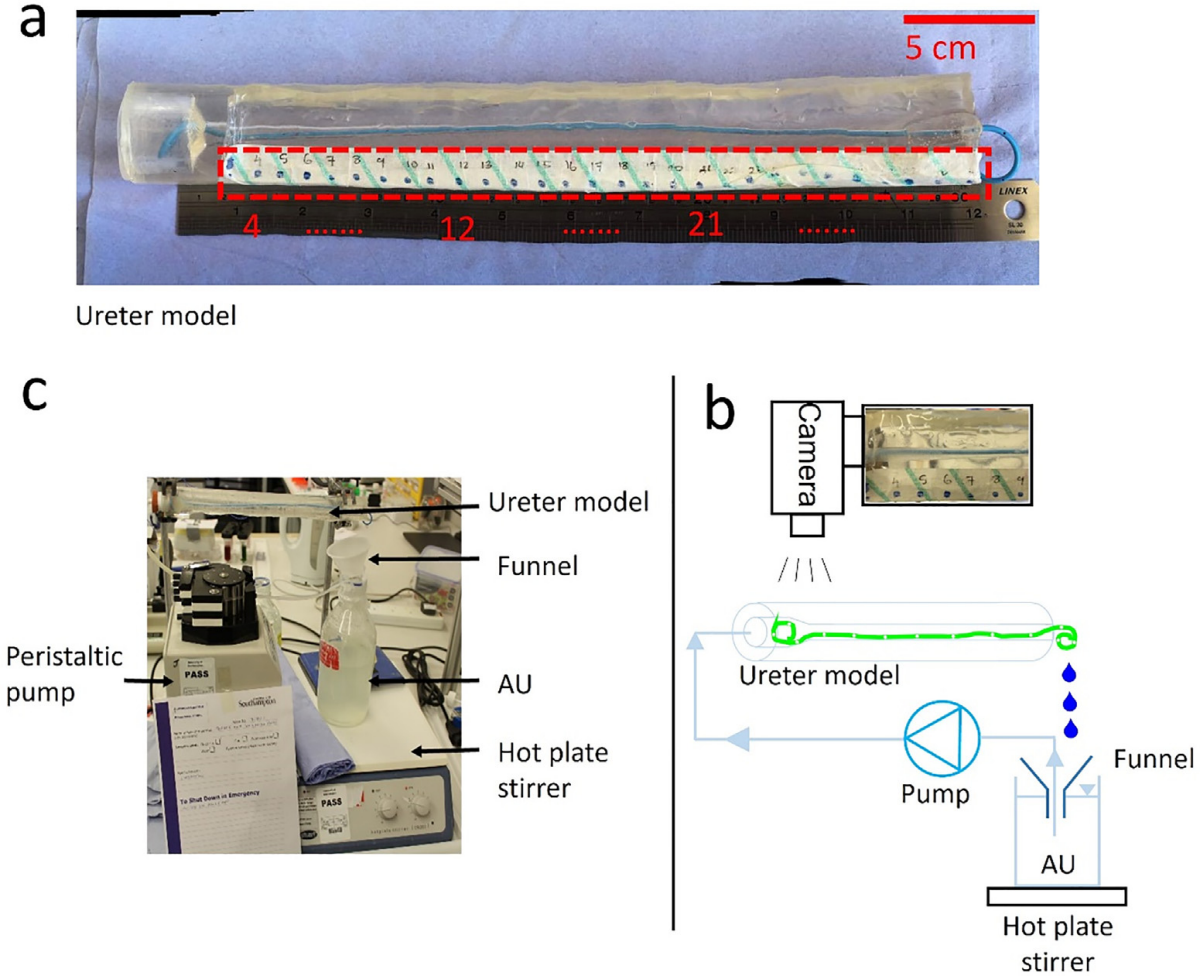
(a) Photograph of the in vitro ureter model. A more detailed description of the model is provided in Ref. 16. The red dashed box indicates the location of the tape used for labeling positions along the model. (b) Experimental setup adopted in this study to carry out in vitro particle-accumulation tests. It included a reservoir containing supersaturated artificial urine (AU), a peristaltic pump (Minipuls3, Gilson), a funnel, a hot plate stirrer (Stuart Hot Plate Stirrer), the ureter model (with the stent shown in green), and a camera (EOS 600D, Canon, Japan). A photograph of the overall setup is also reported in (c).

As discussed earlier, a primary aim of the study was to evaluate whether there exists a correlation between wall shear stress (WSS) and particle accumulation in a three-dimensional model of the stented ureter. Computational fluid dynamic (CFD) simulations were initially carried out to investigate the flow field within the unoccluded stented ureter, where the stent architecture was reconstructed from a commercially available double-J stent (model Universa, Cook Medical, USA) characterized by a wall thickness of 0.5 mm, a length of 28.5 mm (excluding coils), an internal diameter of 1.5 mm, and a total number of 42 side-holes with an average spacing between side-holes of about 7.5 mm [see Figs. 7(a) and 7(b)]. *In vitro* experiments were subsequently carried out on this model to analyze the accumulation of crystals at regions of interest along the stent with a particular focus on side-holes. The experimental particle-accumulation data were then correlated with the numerical WSS values. Once this correlation was established using the unoccluded model, the developed numerical platform was employed as a predictive tool to evaluate stent performance in the presence of a proximal occlusion of the ureter lumen.

### Numerical simulation of the flow field in the stented ureter

CFD simulations were performed to investigate the flow field within the stented ureter, and the computational model was defined in such a way to replicate the experimental conditions. A computer-aided design (CAD) drawing of the ureter model was initially constructed using Autodesk® Inventor Pro 2018 (Autodesk, USA). The fluidic domain was derived by subtracting the stent model from the urinary system model, which was then converted to a format compatible with ANSYS Workbench 18.1 (Ansys, Inc., USA). In order to spatially resolve the wall shear stress (WSS) distribution over the stent surface, the fluidic domain was meshed using tetrahedral volumes (i.e., 25 695 349 and 27 308 086 elements were used for obstructed and unobstructed stented urinary system models, respectively). The minimum and maximum mesh element sizes were set to 0.001 and 0.1 mm (edge length), respectively, based on a compromise between computational cost and solution stability [see Fig. 7(d)]. The inlet volumetric flow rate was set to 1 ml/min, consistently with previous studies,^19,31^ while atmospheric pressure was imposed at the outlet. A no-slip boundary condition was set at the ureter and stent surfaces. The ureter walls were assumed to be rigid, consistently with a study by Gomez-Blanco *et al.*^32^ that demonstrated a negligible mechanical interaction between the stented ureter and urine. The model was assumed to be horizontal (i.e., the gravitational acceleration vector was in the direction of −*x*), as shown in Fig. 7(c). The urine flow field was determined by solving for steady-state mass and momentum conservation equations (referred to as Navier–Stokes equations) over the computational domain.

### Experimental quantification of particle accumulation in a stented ureter model

The PDMS ureter model shown in Fig. 8(a) was employed to investigate accumulation of pre-formed crystals within the stented ureter. Additional information about this model (including photographic images) can be found in Ref. 16. A supersaturated artificial urine was employed in order to facilitate formation of crystals. The urine surrogate composition was taken from Ref. 33 with minor modifications, consistently with our previous study.^19^ It specifically comprised: lactic acid (1.1 mmol/l), citric acid (2 mmol/l), sodium bicarbonate (25 mmol/l), urea (170 mmol/l), calcium chloride dihydrate (2.5 mmol/l), sodium chloride (90 mmol/l), magnesium sulfate heptahydrate (2 mmol/l), sodium sulfate decahydrate (10 mmol/l), potassium dihydrogen phosphate (7 mmol/l), and ammonium chloride (25 mmol/l). Its constituents, thus, included salts that are highly prevalent within ureteric stent encrustations.^34^ Based on our previous microfluidic-based studies that utilized the same urine surrogate formulation, it is anticipated that the average crystal size is of approximately 20 *μ*m. A comprehensive physico-chemical characterization of the formed crystals was not carried out in the present study but could be the subject of future investigations. The temperature of the solution during the experiment was kept constant at 37 °C, and the pH was equal to 6.8 (measured at the beginning of the experiment using a pH meter, Accumet™ AE150 pH, Fisher Scientific™, USA). Using a peristaltic pump (Minipuls3, Gilson^®^), a constant average flow rate of 1 ml/min was generated, which delivered the artificial urine from a reservoir into the stented ureter model in a closed loop configuration [see Figs. 8(b) and 8(c)]. The reservoir was placed on a hot plate stirrer (Stuart^®^ Hot Plate Stirrer) in order to maintain a constant temperature and prevent crystals sedimentation. Crystal accumulation in the model was captured by taking optical images with a CCD camera (EOS 600D, Canon^®^, Japan) placed above the model. Images were acquired at fixed locations (defined by the location of side-holes) along the ureter model [as shown in Fig. 8(a)]. Prior to image acquisition, particular attention was given to make sure that the side-hole was in-focus, and the alignment between the camera and side-hole was comparable between images. The lighting conditions and distance between camera and ureter model were also consistent across image acquisitions. ImageJ software (NIH, USA) was employed to process the images and quantify the time evolution of particle accumulation at these selected regions of interest. Briefly, the acquired images were initially converted to eight-bit format and subsequently into a black-and-white binary format, where black pixels corresponded to areas occupied by crystals (referred to as “coverage area”). Values of the coverage area are reported as a percentage of the total side-hole area and reported as the average value ± standard deviation of three independent experimental repeats. Due to experimental limitations of the image acquisition method employed in this study, it was not possible to determine particle accumulation at each individual side-hole of the stent in an accurate and repeatable way. Therefore, only a selection of side-holes was analyzed, which were located at different regions of interest along the ureter model, i.e., close to the junction between kidney pelvis and ureter (holes 6 and 7), at the first narrowing of the ureter (hole 15), and in proximity to the distal narrowing of the ureter (holes 37 and 38).

### Procedures of the study

In this study, both CFD simulations and experiments were performed to investigate the correlation between WSS and particle accumulation within a full-scale stented ureter model. Numerical results were analyzed at two different spatial scales: global (i.e., along the entire model, from the UPJ to UVJ) and local [i.e., at side-holes of the stent, from hole 1 to hole 42; see Fig. 8(a)]. Numerical simulations, thus, allowed a determination of the magnitude of fluid velocity and wall shear stress at different locations within the model. Where relevant, average values were determined over three spatial domains of the model: proximal (holes 1–8), middle (holes 9–22), and distal (holes 23–42). In a first part of the study, both simulations and experiments were carried out on the unobstructed stented ureter model. In a second part of the study, the numerical model was employed to investigate the flow field within the obstructed stented ureter model.

## SUPPLEMENTARY MATERIAL

See the supplementary material for numerical values of the maximum velocity magnitude and mean WSS at selected side-holes (Tables S1–S5) and box plots showing the distribution of WSS values over the internal and external surface of the stent (Figs. S1–S4).

## Data Availability

The data that support the findings of this study are available from the corresponding author upon reasonable request.
